# Supplementary material for: Identification of photoperiod-induced specific miRNAs in the adrenal glands of Sunite sheep (Ovis aries)
Source: Front Vet Sci. 2022 Jul 22;9:888207. doi: 10.3389/fvets.2022.888207 (PMC9354845; doi:10.3389/fvets.2022.888207)
Supplement: Supplementary file 1 [file Data_Sheet_1.ZIP › Supplementary file/miRDeep2 file.zip!/miRDeep2 file/Know_Structure/SP42A3/oar-mir-16b.pdf]

miRBase precursor : oar-mir-16b
Total read count : 34700
oar-miR-16b read count : 34648
remaining reads : 52

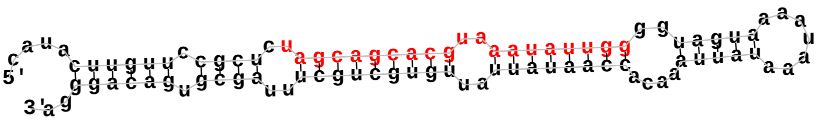

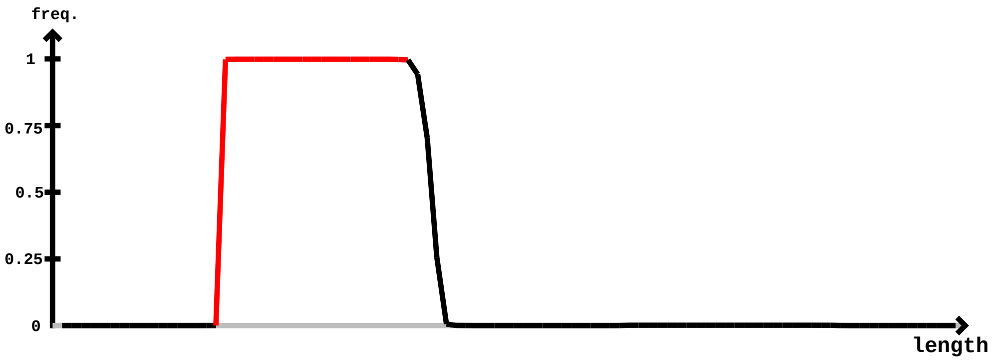


```
                oar-miR-16b
5'- cauacuuguuccgcucuagcagcacguaaauauugggguaguaaaauaaauauuaaacaccaauauuauugugcugcuuuagcgugacaggga   -3'    exp
    ....((((((.((((..(((((((((..((((((((..(((((.......)))))....))))))))..)))))))))..)))).))))))..    reads    mm    sample
    ..............ucuagcagcacguaaauauugg.........................................................    1        0     seq
    ..............ucuagcagcacguaaauauugggg.......................................................    1        0     seq
    ...............cuagcagcacguaaauauuggg........................................................    1        0     seq
    ...............cuagcagcacguaaauauuggggu......................................................    2        0     seq
    ...............cuagcagcacguaaauauuggggua.....................................................    1        0     seq
    ................uagcagcacguaaauau............................................................    1        0     seq
    ................uagcagcacguaaauauu...........................................................    32       0     seq
    ................uagcagcacguaaauauug..........................................................    55       0     seq
    ................uagcagcacguaaauauugg.........................................................    1862     0     seq
    ................uagcagcacguaaauauuggA........................................................    7756     1     seq
    ................uagcagcacguaaauauuggg........................................................    539      0     seq
    ................uagcagcacguaaauauuggAg.......................................................    14040    1     seq
    ................uagcagcacguaaauauugggg.......................................................    1492     0     seq
    ................uagcagcacguaaauauuggggu......................................................    3133     0     seq
    ................uagcagcacguaaauauuggAgu......................................................    5526     1     seq
    ................uagcagcacguaaauauuggggua.....................................................    180      0     seq
    ................uagcagcacguaaauauugggguag....................................................    7        0     seq
    ................uagcagcacguaaauauugggguagu...................................................    7        0     seq
    ................uagcagcacguaaauauugggguaguaaa................................................    1        0     seq
    .................agcagcacguaaauauuggA........................................................    2        1     seq
    .................agcagcacguaaauauuggg........................................................    1        0     seq
    .................agcagcacguaaauauuggAg.......................................................    3        1     seq
    .................agcagcacguaaauauugggg.......................................................    2        0     seq
    .................agcagcacguaaauauuggggu......................................................    3        0     seq
    .................agcagcacguaaauauuggAgu......................................................    3        1     seq
    .................agcagcacguaaauauuggggua.....................................................    1        0     seq
    ..................gcagcacguaaauauuggA........................................................    1        1     seq
    ..................gcagcacguaaauauuggAg.......................................................    1        1     seq
    ..................gcagcacguaaauauuggggu......................................................    1        0     seq
    ..................gcagcacguaaauauugggguag....................................................    1        0     seq
    .........................................................caccaauauuauugugcugcu...............    2        0     seq
    .........................................................caccaauauuauugugcugcuuu.............    3        0     seq
    ..........................................................accaauauuauugugcugcu...............    2        0     seq
    ..........................................................accaauauuauugugcugcuu..............    3        0     seq
    ..........................................................accaauauuauugugcugcuuu.............    24       0     seq
```

oar-miR-16b

```
cauacuuguuccgcucuagcagcacguaaauauuggggguaguaaaauaaauauuaaacaccaauauuauugugcugcuuuagcgugacaggga
..........................................................accaauauuauugugcugcuuua............    7          0          seq
...........................................................ccaauauuauugugcugcuuu.............    2          0          seq
...........................................................ccaauauuauugugcugcuuua............    1          0          seq
```
